# Genomic and phenotypic profiles of two Brazilian breast cancer cell lines derived from primary human tumors

**DOI:** 10.3892/or.2013.2284

**Published:** 2013-02-12

**Authors:** NATÁSSIA C.R. CORRÊA, HELLEN KUASNE, JERUSA A.Q.A. FARIA, CIÇA C.S. SEIXAS, IRIA G.D. SANTOS, FRANCINE B. ABREU, SUELY NONOGAKI, RAFAEL M. ROCHA, GERLUZA APARECIDA BORGES SILVA, HELENICE GOBBI, SILVIA R. ROGATTO, ALFREDO M. GOES, DAWIDSON A. GOMES

**Affiliations:** 1 Department of Biochemistry and Immunology, Federal University of Minas Gerais, Belo Horizonte; 2 Department of Biological Sciences, State University of Londrina, Londrina; 3 Department of Morphology, Federal University of Minas Gerais, Belo Horizonte; 4 NeoGene Laboratory, CIPE; 5 Department of Anatomic Pathology, A.C. Camargo Hospital, São Paulo; 6 Department of Anatomic Pathology, Federal University of Minas Gerais, Belo Horizonte; 7 Department of Urology, School of Medicine, Paulista State University, Botucatu, Brazil

**Keywords:** breast cancer, primary tumor, cell line, tumor xenograft, immunohistochemistry, array comparative genomic hybridization

## Abstract

Breast cancer is the most common type of cancer among women worldwide. Research using breast cancer cell lines derived from primary tumors may provide valuable additional knowledge regarding this type of cancer. Therefore, the aim of this study was to investigate the phenotypic profiles of MACL-1 and MGSO-3, the only Brazilian breast cancer cell lines available for comparative studies. We evaluated the presence of hormone receptors, proliferation, differentiation and stem cell markers, using immunohistochemical staining of the primary tumor, cultured cells and xenografts implanted in immunodeficient mice. We also investigated the ability of the cell lines to form colonies and copy number alterations by array comparative genomic hybridization. Histopathological analysis showed that the invasive primary tumor from which the MACL-1 cell line was derived, was a luminal A subtype carcinoma, while the ductal carcinoma *in situ* (DCIS) that gave rise to the MGSO-3 cell line was a HER2 subtype tumor, both showing different proliferation levels. The cell lines and the tumor xenografts in mice preserved their high proliferative potential, but did not maintain the expression of the other markers assessed. This shift in expression may be due to the selection of an ‘establishment’ phenotype *in vitro*. Whole-genome DNA evaluation showed a large amount of copy number alterations (CNAs) in the two cell lines. These findings render MACL-1 and MGSO-3 the first characterized Brazilian breast cancer cell lines to be potentially used for comparative research.

## Introduction

Breast cancer is a leading cause of death worldwide and represents the primary cause of mortality among women in Brazil (1). Breast tumors are conventionally classified based on prognostic factors, including histological type and grade, proliferation index and angiolymphatic invasion. The St. Gallen Consensus, the American Society of Clinical Oncology (ASCO) and the College of American Pathologists (CAP) also state the evaluation of estrogen receptor (ER), progesterone receptor (PR) and human epidermal growth factor receptor 2 (HER2) status for the prognosis and recommendation of adjuvant therapy (2–4).

Although well-established as prognostic and diagnostic tools, information provided by classical pathological evaluation still fails to predict, with accuracy, the patient’s clinical progression. Thus, the genetic and transcriptional diversity of tumor cells are receiving considerable attention, as they may represent the primary cause of unpredictable tumor behavior and the failure of certain currently used treatments. In their pioneering study, Perou *et al*(5), identified a correlation between histopathological findings and the gene expression profile of various types of breast tumor, correlating classic immunohistochemistry (IHC) and cDNA microarrays. Theirs and subsequent studies (6–8) defined novel molecular subtypes of breast tumors, including luminal A, luminal B, HER2, basal and, more recently, the claudin-low subtype (9).

Subsequently, using an experimental approach similar to that used in previous studies, Kao *et al*(10) applied molecular profile classification to known breast cancer cell lines. Many of the cell lines investigated (MCF-7 or MDA-MB-231) were obtained from metastatic tumors, and are frequently used as breast cancer models. However, metastasis-derived cells have already undergone crucial stages in tumor progression, including the development of invasive capability, cellular adhesion to other organism sites and adaptation to a new environment. Therefore, although widely used, these cell lines do not represent the cells present in primary tumors.

The use of breast cancer cell lines derived from primary tumors as *in vitro* models has rarely been reported and may offer relevant data regarding this type of cancer, increasing the knowledge provided by metastasis-derived cell research. To further understand breast cancer in its initial stages, we investigated the MACL-1 and MGSO-3 breast cancer cell lines previously derived from primary human tumors in our laboratory (11). Correa *et al* characterized these cells lines as authentic tumor and immortalized cell lines through serial passages, loss of contact inhibition, telomerase activity (to confirm immortalization), ability to assemble colonies on agar plates and formation of tumors in immunodeficient mice (11). Moreover, these cells present the differential expression of genes and surface molecules, such as *MUC1* and GAPDH (12), and resistance to γ-irradiation (13).

To gain better understanding of these cell lines, this study evaluated the phenotypic markers from the MACL-1 and MGSO-3 cell lines in comparison to primary tumors and xenograft implants in immunodeficient mice, developed from these cell lines using IHC. Additionally, copy number alterations (CNAs) were evaluated using array comparative genomic hybridization (aCGH). These findings render MACL-1 and MGSO-3 the first characterized breast cancer cell lines to potentially be used for comparative research with other established breast cancer cell lines.

## Materials and methods

### Cell culture

The MACL-1 and MGSO-3 cell lines were previously derived from breast tumor tissue in our laboratory [Correa *et al*(11)]. The cells were grown in Dulbecco’s modified Eagle’s medium (DMEM, Sigma-Aldrich, St. Louis, MO, USA) supplemented with 10% fetal bovine serum (FBS; Sigma-Aldrich) and penicillin/streptomycin (100 U/ml; Life Technologies, Carlsbad, CA, USA) at 37°C in an atmosphere of 5% CO_2_.

### Xenotransplants

Pathogen-free BALB/c.Cg-*Foxn1^nu^*/ AnNTacUnib mice (age, 6–8 weeks) were housed in filter-top cages, and sterile water and food were provided *ad libitum*. The manipulations were conducted aseptically inside a laminar flow hood. One million MACL-1 and MGSO-3 cells were diluted in phosphate buffer and injected subcutaneously between the scapulae of each animal, as described in our previous study (11). The mice were examined for tumor growth every 3 days. When the tumors reached 10 mm in size, the mice were sacrificed and the tumor was dissected for histological examination. Animal experiments were approved by the Animal Use Ethics Committee of the Federal University of Minas Gerais (Belo Horizonte, Brazil).

### Histopathological analysis

Primary tumors were obtained from 2 breast cancer samples obtained from 2 patients (patients 1 and 2) who had presented at Santa Casa de Misericórdia Hospital in Belo Horizonte, Brazil. Samples were routinely processed, embedded in paraffin and 4-μm-thick sections were cut and stained with hematoxylin and eosin (H&E) to evaluate tumor morphology and grade. To evaluate tumor xenografts, the animals were sacrificed and the tumors were excised and fixed in 4% buffered formaldehyde for 24–48 h. Tumor fragments were then rinsed with phosphate buffer, dehydrated in a series of graded ethanol washes and embedded in paraffin. To compare the MACL-1 and MGSO-3 cells grown *in vitro* with tumors grown *in vivo* and primary tumors, the cells were cultured in chamber slides (Lab-TekII, Thermo Fisher Scientific Inc., Waltham, MA, USA). Subsequent to attaining confluence, the cells were fixed with buffered formalin for 1–2 min, washed with phosphate buffer, and stored in this solution until immunohistochemical staining was performed. This study was approved by the institutional Human Ethics Committee (ETIC 03120203000).

### Immunohistochemical analysis

Immunohistochemical analysis was performed using the antibodies shown in Table I(2,3,5,14–16). Sections were deparaffinized using xylene and rehydrated in a series of decreasing concentrations of ethanol solutions. Heat-induced epitope retrieval was then carried out in citrate buffer (sodium citrate, 10 mM; pH 6.0) in a pressure cooker for 4 min at full pressure. Subsequent to cooling, endogenous peroxidase was blocked using a 3% hydrogen peroxide solution for 20 min. The slides were then washed with phosphate buffer solution (10 mM; pH 7.4) and incubated with primary antibodies for 20–30 min or overnight at 4°C and washed 3 times with phosphate buffer. The slides were subsequently incubated using the Advance HRP (Dako, Carpinteria, CA, USA) or MACH 4 Universal HRP-Polymer (Biocare Medical, Concord, CA, USA) detection systems, according to the respective manufacturer’s instructions. The slides were washed 3 times with phosphate buffer and the colored reaction product was developed using 3,3-diaminobenzidine tetrahydrochloride (DAB; Dako) as a substrate for 1 min, while nuclear contrast was achieved using Harris hematoxylin counterstaining. Paraffin sections from the original primary tumors and xenografts were examined using the same procedure. ER and PR staining were evaluated using the Allred scoring system (2). HER2 staining was evaluated as recommended by the CAP/ASCO guidelines (3). Ki-67 was evaluated as the percentage of staining. Qualitative analyses (positivity/negativity) were carried out for the remaining antibodies in the absence of any current official recommendations. Negative controls were obtained by omitting primary antibodies. Heat-induced epitope retrieval was omitted for cultured cells and sections stained for HER2 (clone CB11).

### Clonogenic assay

Cell survival was measured using clonogenic assay (17). Briefly, 900 cells were seeded in 10-cm^2^ plates and incubated for 10 days. Colonies were stained using a mixture of 6.0% glutaraldehyde and 0.5% crystal violet, and then rinsed with water. Colonies with >50 cells were counted as survivors. Surviving fractions were normalized by the plating efficiency of MDA-MB-231 cells. Statistical analysis was carried out using GraphPad Prism 5 software (GraphPad Software, Inc., La Jolla, CA, USA) using one-way ANOVA and Duncan’s post-test. P<0.05 was considered to indicate a statistically significant difference.

### aCGH

Genomic DNA from MACL-1 and MGSO-3 cell lineages was obtained using SDS/proteinase K digestion, followed by phenol/chloroform extraction and ethanol precipitation (18) and treatment with 20 μg/ml RNase A (Sigma-Aldrich). CNAs were evaluated in the MACL-1 and MGSO-3 cell lines using the high-resolution SurePrint G3 Human CGH Microarray kit, 4×180K (Agilent Technologies, Santa Clara, CA, USA). A female genomic DNA control sample (Promega, Fitchburg, WI, USA) was used as the reference. Test and reference DNA were fluorescently labeled using the Agilent Genomic DNA Enzymatic Labeling kit (Agilent Technologies). Experiments were performed in duplicate by swapping dyes between the test and control samples to reduce analytic errors resulting from labeling and hybridization. Subsequent to slide scanning (Agilent DNA Scanner, at 5-μm resolution), image data were extracted and normalized using Feature Extraction 10.1.1.1 software (Agilent Technologies). The array-based CGH data were analyzed using the Nexus Copy Number software version 6.0 (BioDiscovery, Hawthorne, CA, USA) with a FASST2 segmentation algorithm, responsible for the detection of statistically significant CNAs, a sensitivity threshold of 1.00E-6, 3 consecutive probes, and a log_2_ ≤-0.13 and ≥+0.3 for the determination of a loss or gain region, respectively.

## Results and Discussion

### Immunohistochemistry

**Figure 1:**
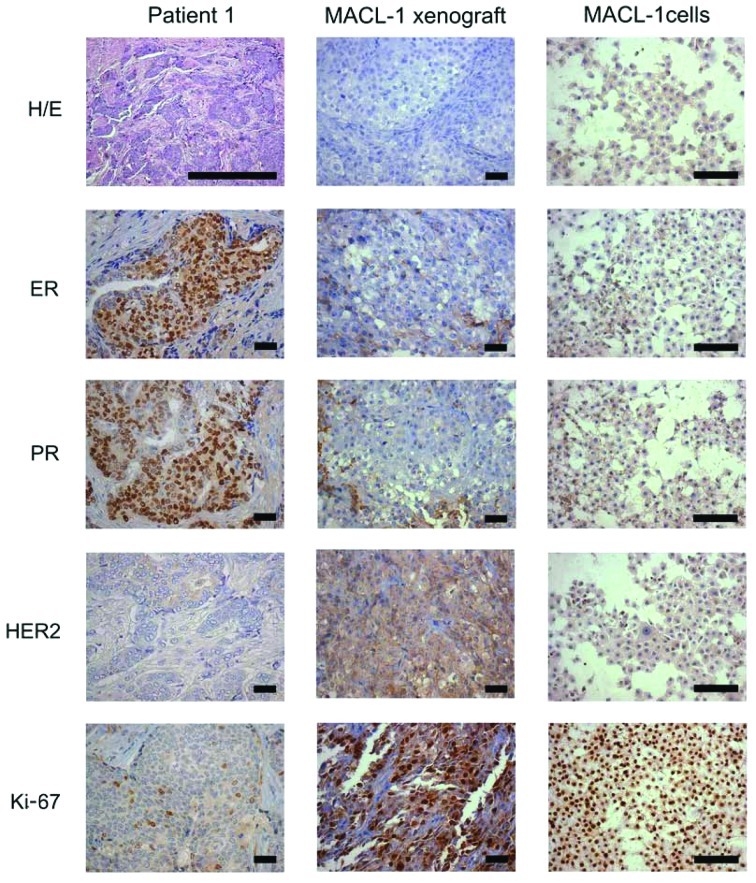
Immunohistochemical profiles of ER, PR, HER2 and Ki-67 markers in the tumor sample from patient 1 (Patient 1); MACL-1-derived tumor xenograft in immunodeficient mouse (MACL-1 xenograft) and MACL-1-cultured cell line (MACL-1 cells). Scale bar, 100 μm.

The breast tumor sample from patient 1 exhibited an invasive ductal carcinoma morphology that may be sub-classified as a luminal A subtype carcinoma (ER/PR-positive and HER2-negative) (Fig. 1). Tumors associated with this subtype are known to be less aggressive and have improved prognosis in patients (6). Moreover, the tumor from patient 1 had a low mitotic grade (<25%), as demonstrated using H&E-stained slides and Ki-67 staining.

**Figure 2:**
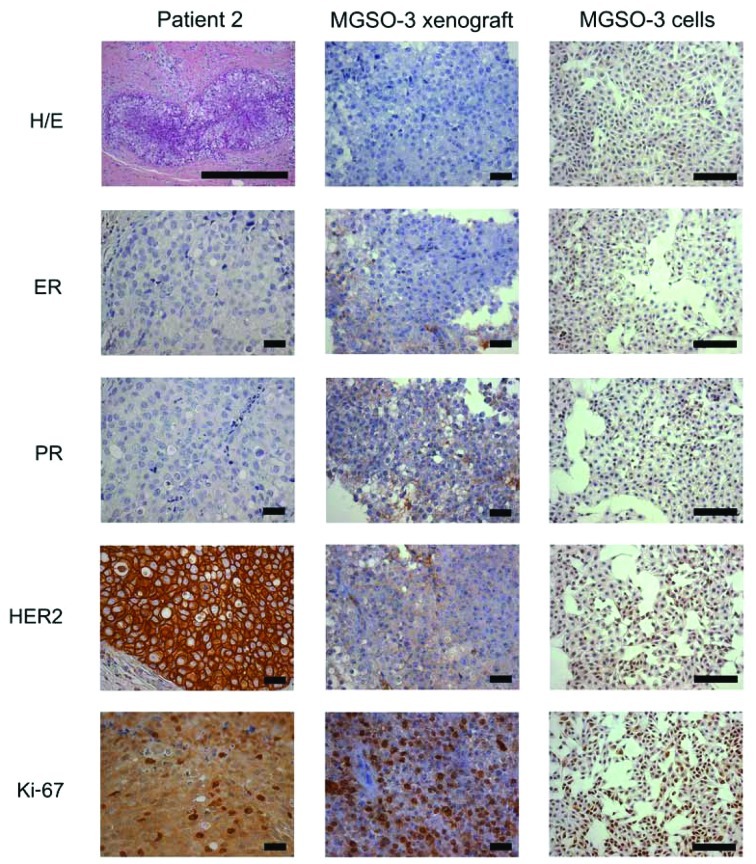
Immunohistochemical profiles of ER, PR, HER2 and Ki-67 markers in the tumor sample from patient 2 (Patient 2); MGSO-3-derived xenograft tumor in immunodeficient mouse (MGSO-3 xenograft) and MGSO-3-cultured cell line (MGSO-3 cells). Scale bar, 100 μm.

Conversely, the breast tumor sample from patient 2 was considered to be a ductal carcinoma *in situ* (DCIS) and presented with a HER2 subtype profile, given that the tumor was negative for ER/PR staining and showed strong HER2 staining (3+) (Fig. 2). Breast tumors of the HER2 subtype have a worse prognosis and comprise some of the most aggressive tumors (6). Additionally, this primary tumor had a high mitotic index (>25%), as demonstrated by H&E and Ki-67-stained slides.

**Figure 3:**
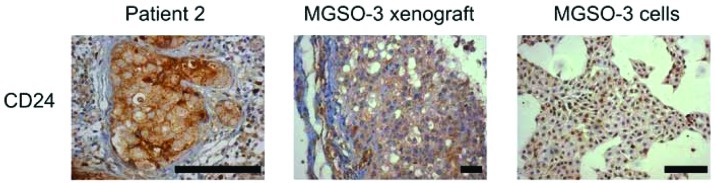
Immunohistochemical staining of CD24 in the tumor sample from patient 2 (Patient 2); MGSO-3-derived xenograft tumor in immunodeficient mouse (MGSO-3 xenograft) and MGSO-3-cultured cell line (MGSO-3 cells). Scale bar, 100 μm.

The tumor sample from patient 2 also showed marked CD24 staining, although MGSO-3 cultured cells and xenografts from these cells were not stained using this marker (Fig. 3). CD24 is a mucin-like adhesion molecule expressed at multiple stages of B-cell development. This protein increases metastatic potential in tumors since it is a ligand of P-selectin, an adhesion receptor of endothelial cells and platelets (19), and has been implicated as an indicator of worse survival prognosis in breast cancer patients (20). Reports that breast cancer stem cells have the CD44^+^/CD24^−^ phenotype, as shown in the study by Al Hajj *et al*(15), are inconsistent with the metastatic role of CD24. Nonetheless, the metastasis process is biologically distinct from that of tumor growth in cancer stem cells, explaining the presence or absence of this marker at diverse stages of breast cancer progression (21).

CD24 staining of the primary tumor of patient 2 may be indicative of a carcinoma that, albeit non-invasive, is associated with tumor progression of a more aggressive phenotype, corresponding to its HER2 subtype classification.

Despite displaying a high mitotic index, the MACL-1 and MGSO-3 cells and their derivative tumor xenografts in the immunodeficient mice did not display ER, PR or HER2 staining. The basal phenotype (Ck5 and EGFR) and the breast cancer stem cell markers (CD44, CD24 and CD133) were absent in the primary tumor and cultured cells of patient 1, as well as the xenografts derived from the 2 cell lines (Table II).

The *in vitro* establishment of cells derived from primary tumors is a rare event, occurring in relatively few attempts (22) and may thus require selection for an ‘*in vitro* establishment’ phenotype (23). It is possible that, as a result of adaptation to a new environment, MACL-1 and MGSO-3 cells shifted to a more appropriate expression pattern for cell culture conditions. Changes in primary tumor markers in the corresponding cultured cell lines have been reported by Brozova *et al*(24) in breast cancer and by Strojnik *et al*(25) in glioblastoma. Differences in the aCGH profiles of breast cancer (26) and the methylation patterns of multiple types of cancer (27) have also been reported in studies comparing cell lines to their respective primary tumors.

Furthermore, the successful transplantation of MACL-1 and MGSO-3 cells into nude mice is noteworthy, since only 7–20% of these implants are successfully accomplished (28). Specifically, the development of *in vivo* xenografts of tumor cells allows for the testing of novel therapeutic approaches and the study of local invasion and interaction with stroma (28).

### Clonogenic assay

**Figure 4:**
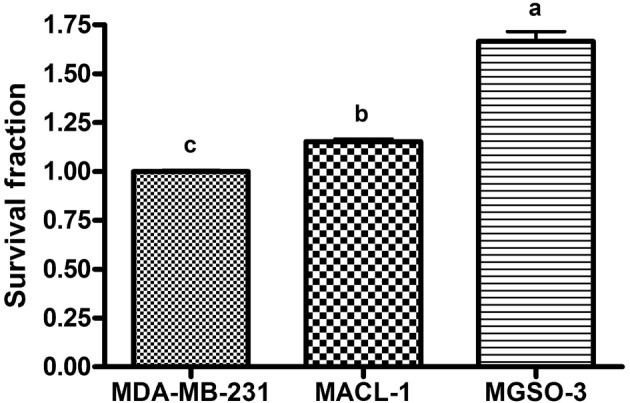
Distinct clonogenic competence of breast cancer cell lines. MACL-1, MGSO-3 and MDA-MB-231 cells were seeded in a 10-cm^2^ dish and incubated for 10 days. Colonies of at least 50 cells were counted as survivors. The mean survival fraction ± standard error of the mean (SEM) of triplicate wells was normalized to that of MDA-MB 231 cells, based on the extent of plating efficiency. Letters a, b and c assign statistical significant difference (Duncan’s post-test, p-value <0.05).

Clonogenic or clonogenic survival assay evaluates the competence of cells to generate a significant number of daughter cells on culture plates after a certain period of time or treatment. The MGSO-3 cell line demonstrated the highest capacity to form colonies after 10 days of incubation, followed by the MACL-1 and MDA-MB-231 lines (Fig. 4). Similar data has been previously reported by Correa *et al*(11), describing the greater proliferative capability of MGSO-3 when compared to MACL-1 cells using a cell doubling time assessment. Additionally, MGSO-3 tumor xenografts in immunodeficient mice were reported to grow more rapidly compared to MACL-1 tumors (11), and the 2 cell lines demonstrated competence to form tumor-like colonies in soft agar. In a subsequent experiment, MGSO-3 cell lines formed the largest and most numerous colonies that were compatible with xenotransplant and culture growth features (11).

### aCGH

**Figure 5:**
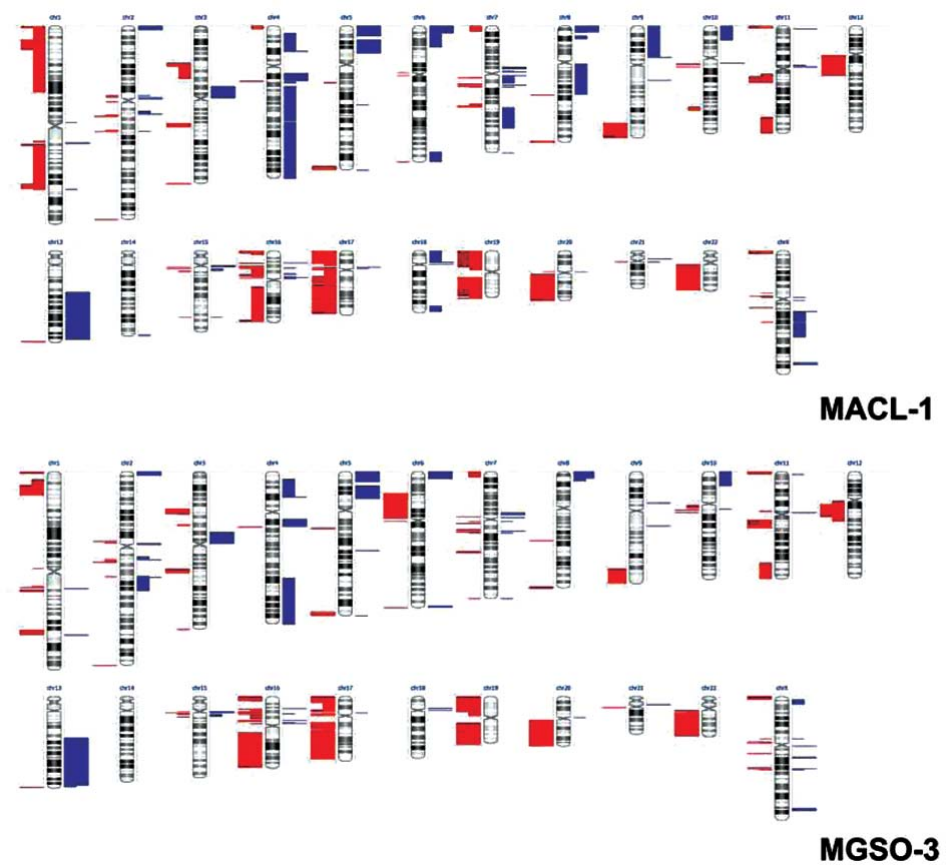
Whole-genome DNA profile of MACL-1 (top panel) and MGSO-3 cells (bottom panel). DNA profiling performed by Human Genome CGH Microarray 4×180K platform hybridization showed large chromosomal alterations in both cell lines. The red and blue bars represent losses and gains, respectively. Double-length bars represent alterations detected on the 2 swapping dye replicates.

Subsequent to slide scanning and data extraction using the Feature Extraction software, aCGH data were analyzed using the Nexus Copy Number software. Fig. 5 displays a whole-genome image derived from the analysis and depicts the extensive chromosomal alterations present in the MACL-1 and MGSO-3 cells, a number of them detected on the 2 dye swapped replicates (represented by double-length bars).

The total CNAs attributed to the MACL-1 and MGSO-3 cell lines were 172.5±30.4 and 166.5±12, respectively. However, when only alterations present on the two replicates and with a p-value <0.05 are considered, 25 and 33 copy number alterations arise for MACL-1 and MGSO-3, respectively.

The complexity of MACL-1 and MGSO-3 genomes has already been observed by our group when we attempted to explore the karyotype profile of these cells through G-banding or DAPI staining (data not shown). This impairment was promptly demonstrated through our aCGH data, which confirmed extremely complex alterations hindering chromosomal mapping through those techniques. MACL-1 and MGSO-3 cell lines displayed common alterations, such as loss of considerable portions of chromosomes 17, 19 and 22 (Table III). Losses on chromosome 17 took place on the following regions: 17q12, 17q12-q21.2, 17q21.2, 17q21.31-q23.1, 17q23.1-q24.1, 17q24.1-q25.2 and 7q25.2-q25.3 (Table III). The MACL-1 and MGSO-3 cells showed significant losses on 17q21.31. Kim *et al*(29) showed that losses on this region are related to prostate cancer and 17q21.31 is known to be completely lost in the PC3 cell line (30). Another important alteration is associated with the 17q12-q21.2 region, where the HER-2 (*ERBB2*) gene is located. The loss of this region could explain the lack of HER-2 expression in MGSO-3 cells and derived xenotransplants from this cell line in nude mice.

For chromosome 19 the affected regions were: 19q13.11-q13.32 in the MACL-1 and MGSO-3 cell lines and 19q13.33-q13.43 in the MGSO-3 cell line (Table III). Loss on 19q13.33–13.43 is a rare finding in human tumors, although it has been described in ovarian cancer cells and gliomas (31,32). The loss of heterozigosity on chromosomes in these types of tumor suggests the location of a tumor suppressor gene, but none has yet been found (31,33,34).

Table III also shows alterations on chromosome 22: 22q11.1-q11.21 and 22q11.21-q13.33. Although our previously karyotyping data showed an apparent intact chromosome 22 (data not shown), Table III shows that chromosome 22 exhibited alterations, which are frequently observed in breast carcinomas (35–38). Previous studies have show frequent allelic loss in this region, but similar to 19q13, a tumor suppressor gene has yet to be confirmed (39). A gene described as important for this region is SMARCB1, also termed IN1. IN1 is considered a tumor suppressor gene and was originally identified in malignant rhabdoid tumors of infancy, and subsequently in medullary carcinomas, sarcomas, myoepithelial carcinomas and chondrosarcomas (40).

Overall the biological processes involved in MACL-1 and MGSO-3 CNAs showed alterations in genes that are engaged in several activities including gene transcription and regulation, cell cycle, signal transduction and metabolic processes. As expected there does not appear to be a concise bias toward a particular biological process.

In conclusion, MACL-1 and MGSO-3 cell lines changed their protein expression profile possibly due to a selection pressure for a more fitted phenotype on cell culture conditions. This phenotypic shift was conserved in tumor xenografts in immunodeficient mice. Despite carrying extensive chromosomal imbalances, these cells maintained a high proliferative ability. To the best of our knowledge, MACL-1 and MGSO-3 are the only Brazilian breast cancer cell lines that could be used for comparative studies with other known breast cancer cell lines.

## Figures and Tables

**Table I:** Primary antibodies, clones, dilution ratios and sources used for immunohistochemical staining.

Antibodies	Clone	Dilution	Source
Estrogen receptor (ER^a^)	6F11	1:100	Neomarkers
Estrogen receptor (ER^b^)	SP1	1:100	Neomarkers
Progesterone receptor (PR^a^)	PgR 312	1:200	Novocastra
Progesterone receptor (PR^b^)	PgR 636	1:400	Dako
HER2^a^	CB11	1:200	Novocastra
HER2^b^	Rabbit polyclonal	1:2,000	Dako
Ki-67	MIB-1	1:800	Dako
CD44	F10-44-2	1:40	Novocastra
CD24	SN3	1:50	Neomarkers
CD133	Rabbit polyclonal	1:100	Abcam
Cytokeratin 5 (CK5)	XM26	1:300	Neomarkers
EGFR	EGFR-25	1:100	Novocastra

Detected using the

a MACH 4 Universal HRP-Polymer and

b Advance HRP detection systems.

**Table II:** Immunohistochemical profiles of the primary tumors of the patients (patients 1 and 2), cultured cell lines (MACL-1 and MGSO-3) and cell line-derived tumor xenografts.

	Patient		Cultured cell line		Tumor xenograft	
			
Antibodies	1	2	MACL-1	MGSO-3	MACL-1	MGSO-3
ER	+	−	−	−	−	−
PR	+	−	−	−	−	−
HER2	−	+	−	−	−	−
Ki-67	+	+	+	+	+	+
CD44	−	−	−	−	−	−
CD24	−	+	−	−	−	−
CD133	−	−	−	−	−	−
CK5	−	−	−	−	−	−
EGFR	−	−	−	−	−	−

**Table III:** Main altered genomic regions on MACL-1 and MGSO-3 cell lines, present on the 2 dye swap replicates, with a p-value <0.05.

Region	Event	Cytoband	Cell line
chr17:0-16531500	Loss	p13.3-p11.2	MACL-1
chr17:31891535-33317141	Loss	q12	MACL-1
chr17:33661605-36347121	Loss	q12	MACL-1 and MGSO-3
chr17:36548604-38591831	Loss	q12-q21.2	MACL-1 and MGSO-3
chr17:38784700-40869210	Loss	q21.2	MACL-1 and MGSO-3
chr17:42143048-57671531	Loss	q21.31-q23.1	MACL-1 and MGSO-3
chr17:57775091-63421974	Loss	q23.1-q24.1	MACL-1 and MGSO-3
chr17:63665720-75057558	Loss	q24.1-q25.2	MACL-1 and MGSO-3
chr17:75269931-78653589	Loss	q25.2-q25.3	MACL-1 and MGSO-3
chr19:32964337-47953667	Loss	q13.11-q13.32	MACL-1 and MGSO-3
chr19:48122394-60078783	Loss	q13.33-q13.43	MGSO-3
chr22:17274835-18691763	Loss	q11.1-q11.21	MACL-1 and MGSO-3
chr22:20247200-49565875	Loss	q11.21-q13.33	MACL-1 and MGSO-3
